# Construction of leaky strains and extracellular production of exogenous proteins in recombinant *Escherichia coli*

**DOI:** 10.1111/1751-7915.12127

**Published:** 2014-04-30

**Authors:** Zhao-Yuan Chen, Jie Cao, Li Xie, Xiao-Fei Li, Zhen-Hai Yu, Wang-Yu Tong

**Affiliations:** Integrated Biotechnology Laboratory, School of Life Sciences, Anhui UniversityHefei, 230601, China

## Abstract

In this study, a strategy of the construction of leaky strains for the extracellular production of target proteins was exploited, in which the genes *mrcA*, *mrcB*, *pal* and *lpp* (as a control) from *Escherichia coli* were knocked out by using single- and/or double-gene deletion methods. Then the recombinant strains for the expression of exogenous target proteins including Trx-hPTH (human parathyroid hormone 1–84 coupled with thioredoxin as a fusion partner) and reteplase were reconstructed to test the secretory efficiency of the leaky strains. Finally, the fermentation experiments of the target proteins from these recombinant leaky strains were carried out in basic media (Modified R media) and complex media (Terrific Broth media) in flasks or fermenters. The results demonstrated that the resultant leaky strains were genetically stable and had a similar growth profile in the complex media as compared with the original strain, and the secretory levels of target proteins into Modified R media from the strains with double-gene deletion (up to 88.9%/mrcA lpp-pth) are higher than the excretory levels from the strains with single-gene deletion (up to 71.1%/lpp-pth) and the host *E. coli* JM109 (DE3) (near zero). The highest level of extracellular production of Trx-hPTH in fermenters is up to 680 mg l^−1^.

## Introduction

In the past decades, the number of recombinant proteins used for therapeutic applications has increased dramatically. Many of these applications involve cell factors with low molecular weight [e.g. human parathyroid hormone (hPTH)] and diverse proteins [e.g. reteplase (rPA), a thrombolytic agent derived from human tissue plasminogen activator]. As a drug used for treating patients with osteoporosis, hPTH secreted by parathyroid gland is composed of 84 amino acid residues (Vad *et al*., 2005; Möricke *et al*., 2011). rPA with approximately 39 kDa, as an effective drug used in thrombolytic and antiplatelet therapy in peripheral vascular disease, consists of the kringle-2, and protease domains of tissue type plasminogen activator after the kringle-1, finger and growth factor domains are removed (Bode *et al*., 1995; Benenati *et al*., 2001).

A variety of alternative expression systems are being developed and evaluated to produce recombinant protein drugs effectively. *Escherichia coli* is the most commonly used host for the production of recombinant proteins. In order to obtain the target exogenous proteins expressed intracellularly in recombinant *E. coli*, cell disruption is necessary, which often results in the increase of pyrogen level (mainly from the cell membrane composition), the increase of sample impurities and the decrease of protein activities. Particularly, the formation of inclusion body often occurs when the target protein is intracellularly overexpressed. To overcome these problems in the production of exogenous proteins, extracellular secretion of exogenous proteins in recombinant *E. coli* is increasingly becoming an important choice. In large-scale industrial production of exogenous proteins, the extracellular excretion of target proteins can remove the cell disruption step, offer a better environment for protein folding and reduce the risk of intracellular enzyme degradation (Mergulhao *et al*., 2005). Besides, the extracellular secretion of target proteins can improve the recombinant protein yield because the target protein accumulation is not limited in periplasmic or intracellular space (Makrides, 1996; Fu *et al*., 2005).

Normally, extracellular secretion of recombinant proteins is blocked mainly due to the difficulties in protein translocation across the two membranes – the cell membrane and the outer membrane of *E. coli* cells (Koebnik *et al*., 2000; Choi and Lee, 2004). Although periplasmic expression of recombinant proteins can often be achieved with the help of a signal peptide, the available methods to overcome the outer membrane barrier for extracellular production of recombinant proteins are limited. In order to solve this problem, various genetic attempts have been made to facilitate the extracellular secretion of recombinant proteins in *E. coli*, including manipulation of transport pathways (Sugamata and Shiba, 2005), optimization of codon and signal sequence (Takemori *et al*., 2012), fusion expression of carrier protein which can be normally secreted extracellularly (Fernandez *et al*., 2000; Choi and Lee, 2004) and fusion expression of outer membrane protein F (Jeong and Lee, 2002), YebF (Zhang *et al*., 2006) or osmotically inducible protein Y (Qian *et al*., 2008). In addition, the coexpression of lysis-promoting proteins such as bacteriocin release protein (BRP) (van der Wal *et al*., 1995) or colicin E1 lysis protein (Kil) (Robbens *et al*., 1995), as well as the use of wall-less strains (the so-called L-forms) (Gumpert and Hoischen, 1998) have also been reported.

Meanwhile, many fermentation techniques, including changes of culture medium compositions (Fu, 2010), temperature (Rinas and Hoffmann, 2004), aeration and calcium ion (Shokri *et al*., 2003), osmotic pressure and induction conditions (Orr *et al*., 2012), as well as the addition of supplements such as glycine (Yang *et al*., 1998) and Triton X-100 (Fu *et al*., 2005; Fu, 2010), have been explored to achieve the extracellular production of recombinant proteins in *E. coli*. The main disadvantage of the fermentation control for the extracellular production of target proteins is that the fermentation conditions vary greatly with different target proteins.

To overcome the uncertainty of the fermentation conditions, the construction of leaky strains (including the *E. coli* Sec pathway) will become a main alternative to transport periplasmic-directed recombinant proteins into media. Leaky strains can be constructed by knocking out of genes related to the biosynthesis of cell wall and membrane, especially of the outer membrane genes such as *lpp* encoding Braun's lipoprotein (Shin and Chen, 2008) of *E. coli*. A lot of studies on the secretion of recombinant proteins in *E. coli* have been reported (Rinas and Hoffmann, 2004; Nandakumar *et al*., 2006; Xia *et al*., 2008; Ostendorp *et al*., 2009); however, the secretory efficiency into media for most of recombinant proteins was not high. Further studies to improve the extracellular production of the target proteins have become inevitable.

In this study, *E. coli* JM109 (DE3), as a popular host for the expression of recombinant proteins, was selected. The genes *pal* (encoding peptidoglycan-associated outer membrane lipoprotein) (Bernadac *et al*., 1998; Cascales *et al*., 2002), *mrcA* (Ishino *et al*., 1980; Broome-Smith *et al*., 1985) and *mrcB* (encoding peptidoglycan synthetase) (Bertsche *et al*., 2005) from this strain were selected as target genes for genetic manipulation; gene *lpp* (encoding Braun's lipoprotein) (Shin and Chen, 2008) was selected as a control.

Several leaky strains including mrcA, mrcB, pal and lpp (single-gene knock-out) as well as lpp mrcB, mrcA lpp, lpp pal, mrcA pal and mrcB pal (double-gene knock-out) were constructed by inframe deletion method to improve the extracellularly secretory levels of their target proteins. All the strains were tested for the secretory levels of their target proteins. Then, the recombinant strains of *E. coli* for the extracellular production of the two proteins Trx-hPTH and rPA were built up. Further, the fermentation experiments of target proteins in both flasks and fermenters were conducted and the yields of the target proteins inside and outside cells were determined.

## Results

### Construction of single or double-gene deleted mutants

In theory, the disruption of genes *mrcA* (2553 bp) or *mrcB* (2535 bp) (Banzhaf *et al*., 2012), both encoding the peptidoglycan synthetase, or the disruption of gene *pal* (522 bp) (Bernadac *et al*., 1998; Cascales *et al*., 2002) encoding the peptidoglycan-associated outer membrane lipoprotein may cause the deficiencies in the structures of their cell walls and the outer membranes, therefore causing the leakage of the intracellular proteins in *E. coli*. To construct leaky strains for extracellular production of target protein(s), *mrcA*, *mrcB* and *pal* as well as *lpp* (237 bp, as a control) (Shin and Chen, 2008) from *E. coli* JM109 (DE3) were selected as target genes for the genetic modification.

**Fig. 1:**
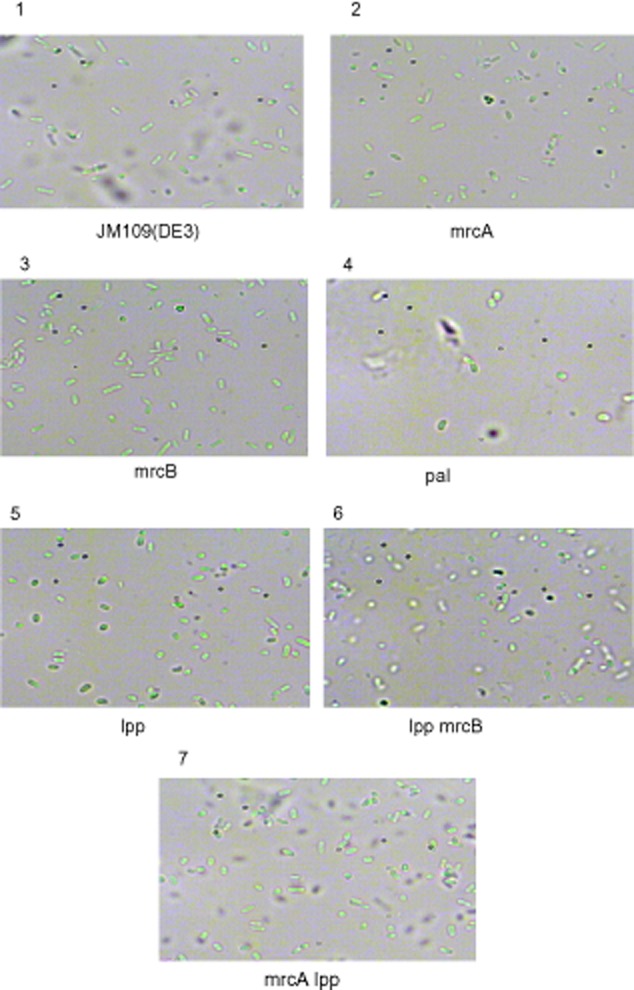
Morphology of the mutants and *E. coli* JM109 (DE3) in microscope CX41RF (Olympus, Tokyo, Japan, 1000× magnification).

Nine mutants named *E. coli* mrcA, mrcB, pal, lpp, lpp mrcB, mrcA lpp, lpp pal, mrcA pal and mrcB pal were obtained by gene manipulation as described in *Experimental procedures*. To identify each mutant, polymerase chain reaction (PCR) amplification of the target genes from leaky mutants of single- or double-gene deletion was carried out by using the corresponding specific ‘verification’ primers (Supporting Information Table S1), and the results of the PCR experiments were illustrated in Supporting Information Fig. S1. The strains mrcA N, mrcB N and lpp N for the construction of double-gene-deleted mutants were also confirmed by sequencing (Supporting Information Table S4). In addition, the morphology of the mutants was observed and compared with their original strain of *E. coli* JM109 (DE3) (Fig. 1). The result demonstrated that the morphological change of strains pal and lpp was significant [shorter and thicker compared with JM109 (DE3)], suggesting the genes *pal* and *lpp* associated with outer membranes are very important in cell morphology.

As shown in Supporting Information Fig. S1, by using corresponding ‘verification’ primers, the colony PCR products of all the mutants, with single or double deletion of genes *mrcA*, *mrcB*, *pal* and *lpp* had the expected band size. Subsequently, the gene sequencing of the PCR products of strains mrcA N, IBL16-02mrcB N and IBL16-04lpp N was carried out (Supporting Information Table S4). The experimental results demonstrated that all the mutants including mrcA, mrcB, pal, lpp, lpp mrcB, mrcA lpp, lpp pal, mrcA pal and mrcB pal have been correctly constructed. However, the mutant of double deletion of gene *mrcB* and *mrcA* was not obtained, suggesting that such a mutation may be lethal to *E. coli* (Denome *et al*., 1999).

### Expression of recombinant protein Trx-hPTH in leaky strains with single-gene deletion

**Fig. 2:**
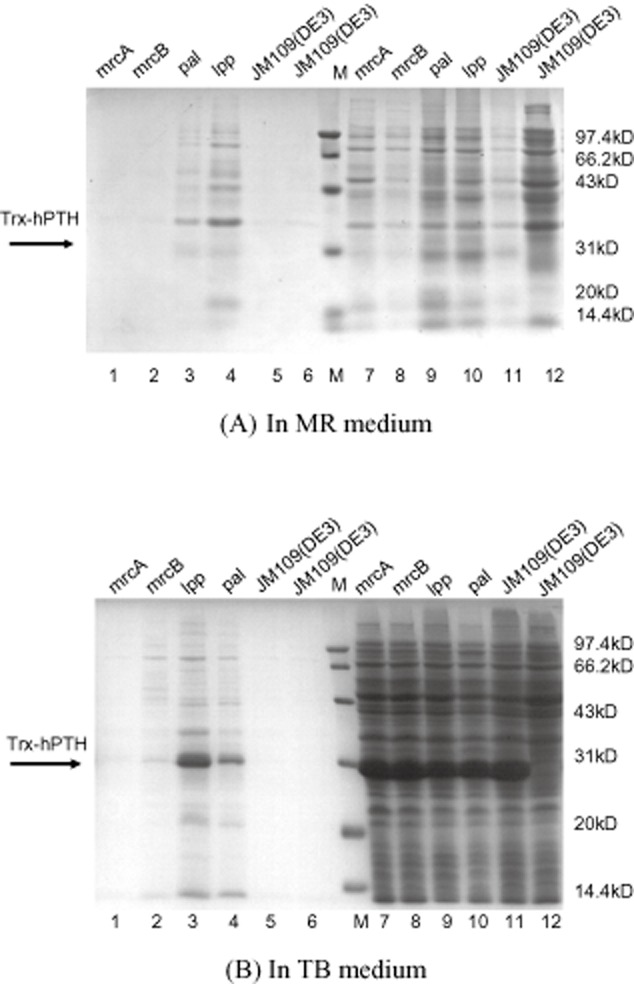
SDS-PAGE analysis of protein samples of leaky strains with single-gene deletion.A. In MR medium; Lane 1, 2, 3 and 4, the extracellular proteins of the recombinant leaky strains mrcA-*pth*, mrcB-*pth*, pal-*pth* and lpp-*pth*; Lane 5 and 6, the extracellular proteins of the strains JM109 (DE3) harbouring pET32a-*pth* and JM109 (DE3); M, protein ladder; Lane 7, 8, 9, 10, 11 and 12, intracellular proteins corresponding to lane 1, 2, 3, 4, 5 and 6.B. In TB medium; Lane 1, 2, 3 and 4, the extracellular proteins of the recombinant leaky strains mrcA-*pth*, mrcB-*pth*, pal-*pth* and lpp-*pth*; Lane 5 and 6, the extracellular proteins of the strains JM109(DE3) harbouring pET32a-*pth* and JM109 (DE3); M, protein ladder; Lane 7, 8, 9, 10, 11 and 12, intracellular soluble proteins corresponding to lane 1, 2, 3, 4, 5 and 6.

To test the secretory efficiency of these leaky strains, the plasmid pET32a-*pth* was first transformed into the leaky strains with single-gene deletion (mrcA, mrcB, pal and lpp). Four recombinant leaky strains mrcA-*pth*, mrcB-*pth*, pal-*pth* and lpp-*pth* were obtained. After fermentation in flasks with Modified R (MR) media, expressed protein samples of each strain were analysed by sodium dodecyl sulfate-polyacrylamide gel electrophoresis (SDS-PAGE) (Fig. 2A) and bicinchoninic acid (BCA) protein assay (Table 1A).

**Table 1A:** Growth analysis and protein detection from the recombinant leaky strains with single-gene deletion in MR medium

Strains	OD_600(A)_^a^	OD_600(B)_^b^	Trx-hPTH^d^		Total proteins^d^	
	
			EPS (mg l^−1^)	IPS (mg l^−1^)	EPS (mg l^−1^)	IPS (mg l^−1^)
mrcA-*pth*	0.92 ± 0.06	2.24 ± 0.06	–c	80 ± 1	960 ± 40	320 ± 5
mrcB-*pth*	0.49 ± 0.04	1.46 ± 0.06	–	50 ± 3	600 ± 30	140 ± 5
pal-*pth*	1.82 ± 0.44 (49 h)	6.24 ± 1.49 (55 h)	220 ± 40	200 ± 50	1190 ± 200	670 ± 180
lpp-*pth*	2.39 ± 0.07	3.73 ± 0.37	420 ± 20	170 ± 20	2040 ± 2	700 ± 60
JM109 (DE3)-*pth*	0.70 ± 0.02	1.79 ± 0.06	–	90 ± 3	750 ± 40	240 ± 10
JM109 (DE3)	2.21 ± 0.01	10.38 ± 0.05	–	–	380 ± 50	2110 ± 70

a. OD_600_ before IPTG induction (∼25 h).

b. Final OD_600_ (∼33 h).

c. Not detected.

d. Protein concentrations are expressed as mean ± SEM of *n* = 2.

As shown in Table 1A and Fig. 2A, approximately 220 mg l**^−1^** Trx-hPTH (51.8% of total Trx-hPTH protein) and 420 mg l**^−1^** Trx-hPTH (71.1% of total soluble Trx-hPTH protein) were obtained from the culture media of pal-*pth* and lpp-*pth* respectively. No Trx-hPTH was obtained from the culture media of mrcA-*pth* and mrcB-*pth*. This observation suggested that the single deletion of gene *pal* encoding peptidoglycan-associated outer membrane lipoprotein or *lpp* encoding Braun's lipoprotein can significantly result in the leakage of the target protein into media, while the single deletion of the gene *mrcA* or *mrcB* encoding peptidoglycan synthetase is not efficient for this leakage under these conditions. The present study also demonstrated that the growth rate of all these strains [especially pal-*pth* incubated for 49 h before isopropyl-β-D-thiogalactopyranoside (IPTG) induction] in MR media was very slow, suggesting nutritional components of MR media are not complete to these strains as compared with Luria–Bertani (LB) or Terrific Broth (TB) media. The growth of strain lpp-*pth* is the best among all the four mutants under the same condition, suggesting that gene *lpp* is not important for the growth of *E. coli*.

To accelerate cell growth and improve the extracellular yield of Trx-hPTH, TB media were selected (Fig. 2B). The final optical density of all these strains was significantly improved after 11 h cultivation. As shown in Supporting Information Table S5, the final optical densities of mrcA-*pth* and mrcB-*pth* were higher than those of pal-*pth* and lpp-*pth*, indicating that the growth limitation of strains pal and lpp can be greatly relieved in rich media. Also, from Fig. 2A and B, we found that the secretory profiles (i.e. the compositions of proteins on SDS-PAGE) of expressed proteins of these leaky strains in both MR media and TB media were similar. Furthermore, the yields of the Trx-hPTH produced by these mutants in TB media are significantly higher than those in MR media, suggesting that the expression efficiency of exogenous proteins are closely related to the cell growth.

It was reported that the supplemented glycine could be incorporated into the nucleotide-activated peptidoglycan precursors (Hammes *et al*., 1973) and thus affected the biosynthesis of peptidoglycan, resulting in the structural defects of cell wall and finally enhancing the extracellular secretion of recombinant proteins in *E. coli* (Li *et al*., 2010). Based on the similar secretory mechanism that partly disrupts the synthesis of cell wall and further indirectly affects outer membrane permeability or both, it is possible for constructing leaky strains with double-gene deletion (genes *mrcA* and *mrcB* for cell wall biosynthesis as well as *pal* and *lpp* for outer membrane biosynthesis) to improve the extracellular secretion of the recombinant proteins (Bertsche *et al*., 2005; Born *et al*., 2006).

### Expression of recombinant protein Trx-hPTH in leaky strains with double-gene deletion

**Fig. 3:**
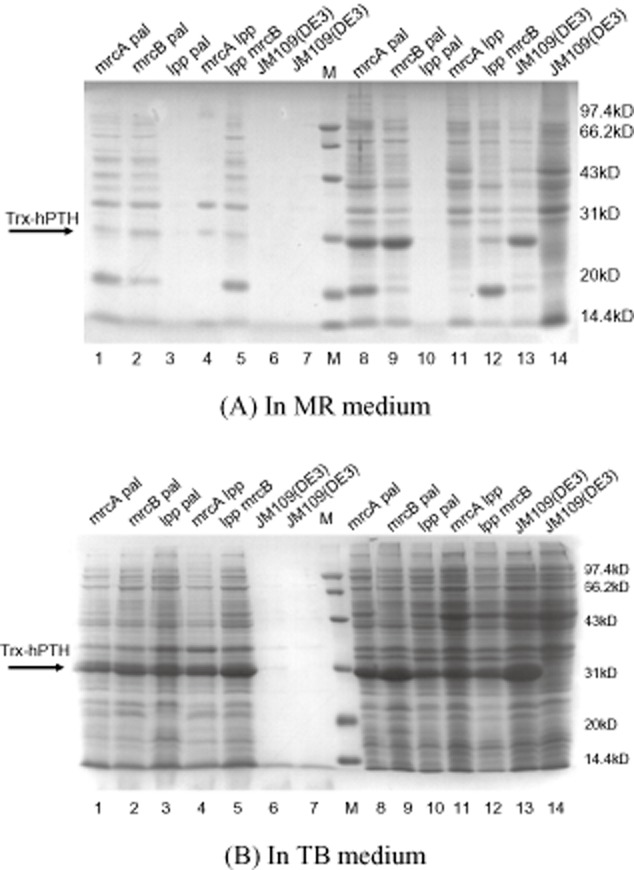
SDS-PAGE analysis of protein samples of leaky strains with double-gene deletion. Lane 1, 2, 3, 4 and 5, the extracellular proteins of the recombinant leaky strains mrcA pal-*pth*, mrcB pal-*pth*, lpp pal-*pth*, mrcA lpp-*pth* and lpp mrcB-*pth*; Lane 6 and 7, the extracellular proteins of the strains JM109 (DE3) harbouring pET32a-*pth* and JM109 (DE3); M, protein ladder; Lane 8, 9, 10, 11, 12, 13 and 14, intracellular proteins corresponding to lane 1, 2, 3, 4, 5, 6 and 7.

Generally speaking, the mutants with double deletion of the genes *mrcA*, *mrcB*, *pal* and *lpp* may result in higher secretory levels of proteins than the mutants with single-gene deletion. To obtain more efficient leaky strains for extracellular production of the target protein hPTH (9.4 kDa) (Prahalad *et al*., 2006), the plasmid pET32a-*pth* expressing Trx-hPTH (∼30 kDa) (Fu *et al*., 2005) was transformed into the leaky strains with double-gene deletion: lpp mrcB, mrcA lpp, lpp pal, mrcA pal and mrcB pal. Five recombinant leaky strains lpp mrcB-*pth*, mrcA lpp-*pth*, lpp pal-*pth*, mrcA pal-*pth* and mrcB pal-*pth* were obtained. After fermentation in flasks with MR media, expressed protein samples of each strain were analysed by SDS-PAGE (Fig. 3) and BCA protein assay (Table 1B).

**Table 1B:** Growth analysis and protein detection from the recombinant leaky strains with double-gene deletion in MR medium

Strains	OD_600(A)_^a^	OD_600(B)_^b^	Trx-hPTH^d^		Total proteins^d^	
	
			EPS (mg l^−1^)	IPS (mg l^−1^)	EPS (mg l^−1^)	IPS (mg l^−1^)
*lpp mrcB-pth*	1.98 ± 0.35	1.98 ± 0.47	150 ± 40	40 ± 10	1370 ± 160	260 ± 80
mrcA lpp-*pth*	0.80 ± 0.002	4.65 ± 0.02	410 ± 1	50 ± 1	1140 ± 20	800 ± 10
lpp pal-*pth*	0.24 ± 0.001	0.25 ± 0.02	90 ± 0	–c	290 ± 1	120 ± 10
mrcA pal-*pth*	2.42 ± 0.14 (14 h)	4.04 ± 0.28 (24 h)	170 ± 10	140 ± 10	1440 ± 100	700 ± 50
*mrcB pal-pth*	1.68 ± 0.05 (13 h)	3.85 ± 0.13 (21 h)	130 ± 10	90 ± 10	1050 ± 100	400 ± 40
JM109 (DE3)-*pth*	1.20 ± 0.01	3.44 ± 0.02	–	140 ± 4	750 ± 10	500 ± 20
JM109 (DE3)	2.99 ± 0.02	15.24 ± 1.62	–	–	310 ± 20	2510 ± 160

a. OD_600_ before IPTG induction (∼16 h).

b. Final OD_600_ (∼25 h).

c. Not detected.

d. Protein concentrations are expressed as mean ± SEM of *n* = 2.

As shown in Table 1B, approximately 150 mg l**^−1^** Trx-hPTH (77.7% of total Trx-hPTH protein), 410 mg l**^−1^** Trx-hPTH (88.9% of total Trx-hPTH protein), 90 mg l**^−1^** Trx-hPTH (concentration of intracellular target protein is undetectable), 170 mg l**^−1^** Trx-hPTH (54.7% of total Trx-hPTH protein) and 130 mg l**^−1^** Trx-hPTH (58.9% of total Trx-hPTH protein) were obtained extracellularly from the double-gene-deleted leaky strains lpp mrcB-*pth*, mrcA lpp-*pth*, lpp pal-*pth*, mrcA pal-*pth* and mrcB pal-*pth* in MR media respectively. The extracellular yields of the target protein from mutants lpp mrcB-*pth*, mrcA lpp-*pth*, mrcA pal-*pth* and mrcB pal-*pth* with double-gene deletion are higher than that from mutant lpp pal-*pth*, suggesting that the deletion mutants of double genes associated with the biosynthesis of outer membrane and cell wall may be more suitable for the extracellular production of target proteins than the deletion mutants of double genes associated with the biosynthesis of outer membrane only.

Judging from the band migration shown in Fig. 3A and B and Supporting Information Fig. S3, it was revealed that the extracellular levels of recombinant protein Trx-hPTH produced by these leaky strains (lpp mrcB-*pth*, mrcA lpp-*pth*, lpp pal-*pth*, mrcA pal-*pth* and mrcB pal-*pth*) in TB media are higher than those in MR media, especially those produced by the strains lpp mrcB-*pth* and mrcA lpp-*pth*.

### Batch cultivation in a fermenter

**Fig. 4:**
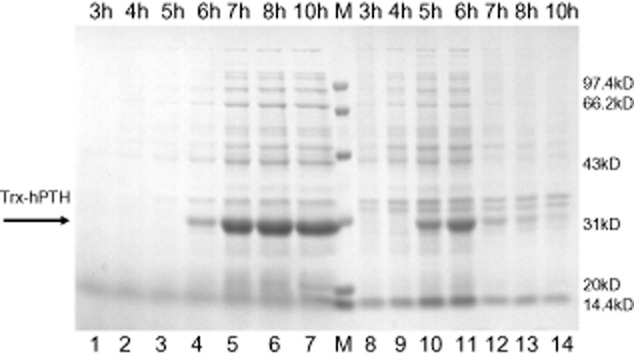
SDS-PAGE analysis of protein samples of lpp mrcB-*pth* during the process of fermentation in TB medium. Lane 1, 2, 3, 4, 5, 6 and 7, the EPSs at 3, 4, 5, 6, 7, 8 and 10 h; M, protein ladder; Lane 8, 9, 10, 11, 12, 13 and 14, intracellular soluble proteins corresponding to lane 1, 2, 3, 4, 5, 6 and 7.

To further test the secretory performances of these mutants in a 5 l fermenter (SY3005B), we chose lpp mrcB-*pth* as a model strain for the extracellular production of Trx-hPTH. The strain was induced with IPTG (0.5 mM) at 4.5 h after inoculation, and the fermentation was continued for 6 h in TB media. The extracellular Trx-hPTH levels increased with the time during the fermentation. Particularly, almost all the Trx-hPTH was transferred into the media after 7 h fermentation and the yield was up to 680 mg l**^−1^** after 10 h cultivation, displaying a better secretory efficiency of Trx-hPTH in fermenter than that in flasks. The SDS-PAGE profiles of Trx-hPTH production in fermenters were shown in Fig. 4. Judging from the bands migration shown in Fig. 4, it appeared that the accumulation of intracellular Trx-hPTH protein (lanes 8 to 14) stopped at 6 h.

### Expression of recombinant protein rPA in leaky strains

In order to test the leakage efficiency of exogenous proteins expressed in cytoplasm from the mutants with inframe single/double deletion of genes *mrcA* and *mrcB* as well as *pal* and *lpp*, the recombinant strains of *E. coli* harbouring pET22b-*rpa* for the expression of the exogenous protein rPA (in cytoplasm owing to no signal peptide) were constructed and then fermented in TB medium. The results demonstrated, as expected, that mutants with inframe single/double deletion of genes *mrcB* and *lpp* caused a little leakage of intracellular proteins (including rPA) into media due to the deficiencies in mutants' cell wall and outer membrane structures, as compared with strain JM109 (DE3)-*rpa* (Supporting Information Fig. S3, Band 1). However, the leakage efficiency for the exogenous protein (rPA) expressed in cytoplasm is much lower than that (Trx-hPTH) expressed in periplasm.

## Discussion

Cell lysis is a main factor affecting the construction and application of leaky strains. To exclude the effect of cell lysis on the extracellular product of exogenous proteins, the growth curves of some leaky mutants were measured (Supporting Information Fig. S4). The mutants of lpp mrcB-*pth* and mrcA lpp-*pth* had normal growth courses like typical *E. coli* (but having relatively low OD_600_ valves at the same time), and no cell lysis occurred within 12 h. Also from Figs 2 and 3, and Supporting Information Figs S2 and S3, we found that the intracellular protein levels for the majority of the samples are significantly higher than the extracellular protein levels, suggesting that the cell lysis does not play a key role in the extracellular production of the recombinant proteins in leaky strains.

The normal cell growth is another important factor in the application of leaky strains. The growth of all leaky stains, as expected, was worse than that of their host *E. coli* JM109 (DE3) (Table 1A). However, the optical density at 600 nm (OD_600_) and intracellular protein sample (IPS) of the recombinant leaky strains, except for mrcB-*pth*, were higher than those of JM109 (DE3)-*pth*. In addition, we found that after 25 h cultivation in MR medium, the OD_600_ of almost all these mutants (lpp mrcB-*pth*, mrcA lpp-*pth*, mrcA pal-*pth* and mrcB pal-*pth*) with double-gene deletion in MR medium was higher than their corresponding mutants (mrcA-*pth*, mrcB-*pth*, pal-*pth* and lpp-*pth*) with single-gene deletion, suggesting that the double deletion of genes *mrcA* or *mrcB* (associated with the biosynthesis of the cell walls of *E. coli*) and genes *pal* or *lpp* (associated with the biosynthesis of the outer membranes of *E. coli*) are more suitable for the growth of *E. coli* than the single deletion of genes *mrcA* or *mrcB* in MR medium. The growth of strain lpp pal-*pth* was retarded in MR medium. The reason for this remained unclear at the present, but it may be due to the removal of two lipoprotein genes related to the outer membrane integrity and cell division cycle (Gerding *et al*., 2007).

When TB medium was used for extracellular protein production, the final optical density of all these strains was improved after 11 h cultivation (Supporting Information Table S6). The final optical density of lpp pal-*pth* was higher than that of mrcA pal-*pth* and mrcB pal-*pth*, suggesting that the growth limitation of Δ*lpp* and Δ*pal* strains can be relieved in TB medium (a complete medium). The further study on their mechanisms is warranted. The present study demonstrated that the extracellular yields of recombinant protein Trx-hPTH from the mutants with double deletion were significantly higher than those from the mutants with single deletion under the same condition (Supporting Information Fig. S2). In addition, our results showed, as expected, that mutants with inframe single/double deletion of genes, *mrcB* and *lpp*, could not cause the efficient leakage of the target protein rPA due to the protein expression in cytoplasm, not in periplasmic space (Supporting Information Fig. S3).

The comparison between the intracellular and extracellular production of Trx-hPTH using mutant lpp mrcB-*pth* as a model strain revealed that the extracellular production of Trx-hPTH continued to rise after the intracellular yield achieved a steady level (Fig. 4). In comparison, the intracellular accumulation of Trx-hPTH began to decrease after 6 h of fermentation. The probable reason is that the increasing accumulation of Trx-hPTH in periplasmic space results in the leaky capability increase of outer membrane of mutant cells. In addition, the extracellular excretion of target proteins can remove the cell disruption step, offer a better environment for protein folding, reduce the risk of intracellular enzyme degradation (Mergulhao *et al*., 2005) and has unlimited periplasmic or intracellular space (Makrides, 1996; Fu *et al*., 2005). Thus, the extracellular yield of target proteins could continuously increase even after the accumulation of intracellular production stops.

In conclusion, there was no apparent cell autolysis during the fermentation process of exogenous proteins. High leaky levels of Trx-hPTH were achieved from lpp mrcB-*pth* (77.7%) and mrcA lpp-*pth* (88.9%) with the double-gene deletion of *lpp* and *mrcB* as well as *mrcA* and *lpp* in MR medium. Among all the mutants with inframe single/double-gene deletion, the extracellular production levels of target proteins in TB medium were much higher than those in MR medium. Particularly, almost all the Trx-hPTH has been transferred into the medium after 7 h fermentation in fermenters. All the results above suggested that double deletion of peptidoglycan synthetase genes and outer membrane genes may increase the outer membrane permeability enough for the leakage of periplasmic protein without affecting the growth of these strains significantly in complex media.

Herein, the main advantage of leaky strains is that no any additive is needed to induce extracellular protein production and the main disadvantage is that the secretory selectivity is not high, suggesting that these genes affect the structure of the outer membrane but do not participate in the active transport of target protein(s) (Mergulhao *et al*., 2005).

## Experimental procedures

### Strains and plasmids and primers

The strain *E. coli* JM109 (DE3) was used as a host for genetic modification. The plasmid pKD46 (Amp^R^) was used as a helper plasmid for increasing the efficiency of homologous recombination, and pCP20 (Cm^R^/Amp^R^) was used for eliminating the resistance marker of the mutants in the construction of leaky strains (Datsenko and Wanner, 2000). The plasmids pET32a-*pth* and pET22b-*rpa*, both constructed without signal sequences, were used for the expression of exogenous proteins Trx-hPTH (a thioredoxin fusion protein used for its residence at the periplasmic space of *E. coli*) (Bayer *et al*., 1987; Fu *et al*., 2005) and rPA (Wang *et al*., 2009) in periplasmic space or in cytoplasm. The primers were designed by Primer Premier 5.0 and verified by Oligo 7.0. All the strains, plasmids, target fragments (TFs) and ‘verification’ primers used in this study are shown in Supporting Information Table S1.

### Media

To activate and select strains, LB medium containing 10 g l**^−1^** tryptone (Oxoid, England), 5 g l**^−1^** yeast extract (Oxoid) and 5 g l**^−1^** NaCl was prepared (1.5% agar added in solid medium). The media were supplemented with ampicillin (Amp, a final concentration of 50 μg ml**^−1^**), kanamycin (Kan, 30 μg ml**^−1^**) or chloramphenicol (Cm, 25 μg ml**^−1^**) as required. To quantitatively detect target proteins, MR medium (a basic medium without proteins) was utilized in this study (Fu *et al*., 2005). After primary screening, TB medium (a complex medium) (Fu *et al*., 2005) was selected for improving the yield of extracellular production of recombinant proteins.

### Gene manipulation

The leaky strains were constructed by using one-step inactivation method (Datsenko and Wanner, 2000; Baba *et al*., 2006). Briefly, the specific TFs with the flanking flippase recognition target sites used for the gene knockout, which spans the chloramphenicol acetyltransferase (*cat*) or kanamycin (*kan*) resistance cassettes, were amplified by a PCR method using corresponding primers shown in Supporting Information Table S1. Plasmid pKD3 (Cm^R^) or pKD4 (Kan^R^) were used as templates. The TFs have 50-nucleotide extensions that are homologous to the adjacent regions of the genomic *mrcB*, *mrcA*, *lpp* and *pal* genes (Supporting Information Table S2).

To construct leaky strains (mrcA, mrcB, pal and lpp) with inframe single deletion of genes *mrcA*, *mrcB*, *pal* and *lpp*, each corresponding TF was electro-transformed (MicroPulser Electroporator, Bio-Rad, Philadelphia, USA) into *E. coli* JM109 (DE3) harbouring pKD46 (Amp^R^) which can express the λ Red recombinase after l-arabinose induction. The resulting mutants were selected on LB agar plates containing 30 μg ml^−1^ Kan or 25 μg ml^−1^ Cm, and subsequently verified by a colony PCR method using the ‘verification’ primers listed in Supporting Information Table S1. In the processing of single deletion of genes, plasmid pKD4 or pKD3 (only for the single deletion of the gene *pal*), primer couples mrcA-F/mrcA-R, mrcB-F/mrcB-R, pal-F/pal-R and lpp-F/lpp-R as well as ‘verification’ primers mrcA-vF/mrcA-vR, mrcB-vF/mrcB-vR, pal-vF/pal-vR and lpp-vF/lpp-vR were correspondingly used.

To construct leaky strains (lpp mrcB, mrcA lpp, lpp pal, mrcA pal and mrcB pal) with inframe double-gene deletion, the antibiotic marker of single-gene-deleted strains was first removed by cloning a helper plasmid pCP20 (Amp^R^, Cm^R^) using competent cell preparation kit GK6031 (Shanghai Generay Biotech, Shanghai, China). The pCP20 contains the gene encoding the flippase recognition target (FLP) recombinase and a temperature-sensitive replication origin that creates thermal induction of FLP synthesis (Datsenko and Wanner, 2000); therefore, the positive colonies losing the resistance to ampicillin, kanamycin or chloramphenicol can be selected under suitable temperatures. More specifically, the positive mutants must be able to grow on the agar plates containing 50 μg ml^−1^ Amp at 30°C and can grow on the same plates without Amp at 39°C due to the thermal induction of FLP synthesis and the thermal blockage of the replication of pCP20. The mutants without antibiotic resistance markers were further identified using the colony PCR and sequencing methods. The procedures for the construction of leaky strains with double deletion of genes were similar to those described above except for the TFs and the original strains. (For more details about the plasmids, primers, resistance markers, TFs as well as the kinship of strains, please see Supporting Information Table S3.)

To test the performance of the leaky strains (Supporting Information Table S1) with single- or double-gene deletion, the pr eviously constructed plasmids (Table 1) expressing Trx-hPTH and rPA were transformed into these strains using the competent cell preparation kit GK6031 (Shanghai Generay Biotech) and then identified by DNA analysis and protein assay described as below. To test the genetic stability of them, the expression analysis of exogenous proteins were conducted in LB medium with the addition of ampicillin after the seventh generation culture of the recombinant leaky strains, and their growth curves were obtained. Finally, the leaky efficiency of target proteins from these strains was tested by fermentation experiments and protein assay.

### Flask fermentation

To prepare inoculums, a single colony of each recombinant leaky strain (Supporting Information Table S3) with expression plasmid from LB plates was inoculated into each 250 ml flask containing 30 ml of MR medium supplemented with ampicillin (50 μg ml^−1^) and incubated in a rotary shaker at 200 r.p.m. at 37°C until the concentration of the strain (OD_600_) was higher than 1.0. The prepared culture was inoculated into a 250 ml flask containing 30 ml MR medium (inoculum size of 1–2%) supplemented with ampicillin (50 μg ml^−1^), and followed by incubation at 37°C in a rotary shaker at 200 r.p.m. After cultivation for 25 h (single-gene mutants) or 16 h (double-gene mutants), IPTG (Sangon Biotech, Shanghai, China) was added into the culture with a final concentration of 0.5 mM to induce the recombinant protein expression and the culture was cultivated for an additional 8–9 h. During the fermentation process, the cell density was determined by measuring the optical density at 600 nm (OD_600_) with a BioPhotometer plus spectrophotometer (Eppendorf, Hamburg, Germany).

To improve the secretory efficiency of target proteins in the leaky strains, complex LB media and TB media were used. To prepare inoculums, a single colony of each recombinant leaky strain (Supporting Information Table S3) with expression plasmid from LB plates was inoculated into each 250 ml flask containing 30 ml of LB medium supplemented with ampicillin (50 μg ml^−1^) and incubated overnight at 37°C in a rotatory shaker at 200 r.p.m. The overnight culture was inoculated into a 250 ml flask containing 30 ml TB medium supplemented with ampicillin (50 μg ml^−1^) to reach an OD_600_ of 0.1, followed by incubation at 37°C in a rotary shaker at 200 r.p.m. After cultivation for 7 h, IPTG (Sangon Biotech) was added into the culture with a final concentration of 0.5 mM to induce recombinant protein expression and the culture was cultivated for an additional 3.5–4 h. During the fermentation process, the cell density was determined by measuring the optical density at 600 nm (OD_600_) with a BioPhotometer plus spectrophotometer (Eppendorf).

### Batch fermentation in a 5 l fermenter

One single colony of *E. coli* lpp mrcB harbouring pET32a-*pth* from a LB plate was inoculated into a 250 ml flask containing 30 ml of LB medium supplemented with 50 μg ml^−1^ ampicillin and then incubated at 37°C in a rotary shaker at 200 r.p.m. The overnight culture was inoculated into a 250 ml flask containing 30 ml of TB medium supplemented with 50 μg ml^−1^ ampicillin and then incubated at 37°C in a rotary shaker at 200 r.p.m. After approximately 8 h cultivation, the culture was inoculated into the bioreactor SY3005B (Shanghai Shiyuan Bio-engineering Equipment, China) (2% inoculum size) containing 3 l TB medium supplemented with 50 μg ml^−1^ ampicillin. The cultivation was conducted at 37°C, initial pH of 7.2, 0.5–1.0 vvm of aeration and 300 r.p.m. of agitation. After 4.5 h cultivation, IPTG was added at a final concentration of 0.5 mM to induce Trx-hPTH expression and the cells were cultured for an additional 6 h. During the cultivation, optical density at 600 nm (OD_600_) of the culture was measured by a BioPhotometer plus spectrophotometer (Eppendorf). Protein samples were prepared and analysed as described below.

### Sample preparation

To prepare samples for the analysis of recombinant proteins, 1 ml of each culture was pelleted by centrifugation at 10 000 r.p.m. for 5 min at room temperature. The resulting supernatant fraction was defined as extracellular protein sample (EPS) and the pellet fraction as IPS. To differentiate IPS further, the pellet was washed twice by phosphate-buffered saline (PBS) (Na_2_HPO_4_·12H_2_O 7.16 g l**^−^**^1^, KH_2_PO_4_ 2.72 g l**^−^**^1^; 0.02 M, pH 8.0), centrifuged at 10 000 r.p.m. for 5 min, resuspended in 1 ml PBS, subjected to ultrasonication and centrifuged at 10 000 r.p.m. for 5 min again. The supernatant fraction collected was defined as intracellular soluble protein sample and the precipitate fraction as intracellular insoluble protein sample. After that, the EPSs and IPSs were analysed by SDS-PAGE (Fu *et al*., 2005). The total protein levels of protein samples were measured by BCA protein assay as described below.

### DNA gel and SDS-PAGE

DNA electrophoresis for PCR product analysis was carried out at 110 V for 30 min (Sambrook and Russel, 2011). Briefly, the samples of PCR products were mixed with 5× loading buffer (Shanghai Generay Biotech, China), and then loaded onto agarose gel (1%) with ethidium bromide. After electrophoresis, the gel was scanned by TANON gel image system 1600 (TANON Science and Technology, Shanghai, China).

SDS-PAGE was run at 110 V for 2 h according to Schägger's publication (Schägger and von Jagow, 1987). Briefly, 40 μl protein sample was mixed with 10 μl Sample Loading Buffer (5×) SD8320 (Sangon Biotech) and boiled at 100°C for 5 min, and then the mixture was loaded onto SDS-PAGE gels (15%) with a volume of 15 μl. After electrophoresis, the gels were stained with Coomassie R250 (Shanghai Solarbio Bioscience & Technology, Shanghai, China) and then scanned by the TANON gel image system 1600. To determine the proportion of target protein in extracellular or IPS, the protein bands were normalized and quantified by a GIS 1D analysis software 4.1.2 (TANON Science & Technology).

### BCA protein assay

The protein levels (including Trx-hPTH and total proteins) of all protein samples were measured by BCA method (protein assay kit-SK3051, Sangon Biotech) using bovine serum albumin (BSA) as standard. Serial dilutions of commercial BSA (Sangon Biotech) were used for constructing a standard curve. Samples with appropriate dilutions were prepared in duplicates, each with 50 μl. After addition of 1 ml working stock AB solution (Sangon Biotech), each sample in a tube was incubated at 37°C for 30 min. After cooling down to the room temperature, the absorbance OD_562_ of each sample was measured by a BioPhotometer plus spectrophotometer (Eppendorf). The mean concentration of each protein sample was determined by using absorbance A_562_ value according to the BSA protein standard curve.

### Authors' contributions

Zhao-Yuan Chen carried out the experiments and interpretation of results and drafted the manuscript. Li Xie, Jie Cao, Xiao-Fei Li, Zhen-Hai Yu and Jie Yu participated in the experiments or interpretation of results. Wang-Yu Tong contributed to study design, interpretation of results and writing of the manuscript.

## Supporting information

**Fig. S1.** Verification of the mutants by colony PCR (Lane M: representing standard marker). (A) Single knockout of gene *mrcA* (*kan*/1700 bp): Lane 1, JM109 (DE3) as a template; Lane 2, 3 and 4, mrcA as a template; (B) Single knockout of gene *mrcB* (*kan*/1700 bp): Lane 1 and 2, JM109 (DE3) as a template; Lane 3 and 4, mrcB as a template; (C) Single knockout of gene *pal* (*cat*/1600 bp): Lane 1 and 2, JM109 (DE3) as a template; Lane 3, 4 and 5, pal as a template; (D) Single knockout of gene *lpp* (*kan*/1800 bp): Lane 1, 2, 3 and 4, lpp N as a template; Lane 5 JM109 (DE3) as a template; Lane 6, 7 and 8, lpp as a template; (E) Double knockout of genes *lpp* and *mrcB* (*cat*/1200 bp) as well as *mrcA* and *lpp* (*kan*/1800 bp); Lane 1 and 6, others, Lane 3, JM109 (DE3) as a template; Lane 4, lpp mrcB (Cm^R^) as a template (mrcB-vF and mrcB-vR as primers); Lane 5, lpp mrcB (Kan^R^) as a template (mrcB-vF and mrcB-vR as primers); Lane 6, lpp N as a template (lpp-vF and lpp-vR as primers); Lane 7, mrcA lpp (Kan^R^) as a template (lpp-vF and lpp-vR as primers); (F) Double knockout of genes *lpp* and *pal* (*kan /*2100 bp); Lane 1 and 2, lpp pal (Kan^R^) as a template (pal-vF and pal-vR as primers); Lane 3 and 4, (Cm^R^) as a template (pal-vF and pal-vR as primers); Lane 5, nothing; lane 6, JM109 (DE3) as a template; (G) Double knockout of genes *pal* and *mrcB* (*kan* /1700 bp) as well as *pal* and *mrcA* (*kan*/1700 bp); Lane 1, 3 and 5, mrcB pal as a template (pal-vF and pal-vR as primers); Lane 2 and 4, false positive colonies as a template (pal-vF and pal-vR as primers); Lane 6, 7, 8 and 9, JM109 (DE3) as a template; Lane 10, mrcA pal as a template (pal-vF and pa-vR as primers).

**Fig. S2.** SDS-PAGE analysis of protein samples in TB medium. Lane 1 and 2, the extracellular proteins of the recombinant leaky strains lpp mrcB with pET32a-*pth*; Lane 3, 4, 5 and 6, the extracellular proteins of the recombinant leaky strains mrcA lpp, lpp, mrcB and mrcA with pET32a-*pth*; Lane 7, the extracellular proteins of the strains JM109 (DE3) harbouring pET32a-*pth*; M, protein ladder; Lane 8, 9, 10, 11, 12, 13 and 14, intracellular soluble proteins corresponding to lane 1, 2, 3, 4, 5, 6 and 7.

**Fig. S3.**-SDS-PAGE analysis of protein samples of lpp mrcB with pET22b-*rpa* in TB medium (M, protein ladder). Lane 1, 2, 3, and 4, the extracellular proteins of the recombinant strains lpp mrcB, mrcB, lpp and JM109 (DE3) harbouring pET22b-*rpa*; Lane 5, 6, 7 and 8, intracellular soluble proteins corresponding to lane 1, 2, 3 and 4; Lane 9, 10, 11 and 12, intracellular insoluble proteins corresponding to lane 1, 2, 3 and 4.

**Fig. S4.**-Growth curve of the strains in flasks in LB medium. (A) ▪, strain lpp mrcB with pET32a-*pth*; •, Strain lpp mrcB harbouring pET32a-*pth* with IPTG induction after four hour cultivation; ▴, Strain mrcA lpp with pET32a-*pth*. (B) ▪, strain mrcB; •, strain JM109 (DE3).

**Table S1.** Strains, plasmid and primers used in this study.

**Table S2.** The sequences of TFs (targeting fragments).

**Table S3.** The plasmids, primers, resistance markers, targeting fragments and hosts used for mutant construct.

**Table S4.** Sequences of the PCR products of the mutants of mrcAN, mrcBN and lppN.

**Table S5.** Growth analysis and protein detection from the recombinant leaky strains with single-gene deletion in TB medium.

**Table S6.** Growth analysis and protein detection from the recombinant leaky strains with double deletion in TB medium.
