# Supplementary material for: Construction of leaky strains and extracellular production of exogenous proteins in recombinant Escherichia coli
Source: Microb Biotechnol. 2014 Apr 30;7(4):360–70. doi: 10.1111/1751-7915.12127 (PMC4241728; doi:10.1111/1751-7915.12127)
Supplement: Supplementary file 1 [file mbt20007-0360-sd1.docx]

Supporting Information

## Construction of leaky strains and extracellular production of exogenous proteins in recombinant *Escherichia coli*

### Running head: Extracellular production of exogenous proteins

Zhao-Yuan Chen, Jie Cao, Li Xie, Xiao-Fei Li, Zhen-hai Yu and Wang-Yu Tong*

Integrated Biotechnology Laboratory, School of Life Sciences, Anhui University, Hefei 230601, China

**Corresponding author:*

*Postal address: Integrated Biotechnology Laboratory, School of Life Sciences, Anhui University, 111 Jiulong Road, Hefei 230601, China;*

*Tel.: +86-0551-63861282;*

*Fax: +86-0551-63861282;*

*E-mail: tongwy@ahu.edu.cn*

**Supporting Information**

Table S1. Strains, plasmid and primers used in this study

| Items | Relevant properties | Note | | |
| --- | --- | --- | --- | --- |
| **Strains** | **Genotype** | **Source** | | |
| *E. coli* JM109 (DE3) (IBL16)* | *end*A1, *rec*A1, *gyr*A96, *thi*, *hsd*R17 (rk^–^, mk^+^), *rel*A1, *sup*E44, λ–, Δ(*lac-pro*AB), [F’, *tra*D36, *pro*AB, *lac*I^q^ZΔM15], lDE3 | Promega | | |
| J M109 (DE3) -pKD46 (IBL16-pKD46)* | J M109 (DE3) harboring pKD46 | In this study | | |
| mrcA (IBL16-01) * | ∆*mrcA*:: *kan* JM109(DE3) strain | In this study | | |
| mrcB (IBL16-02) * | ∆*mrcB*:: *kan* JM109(DE3) strain | In this study | | |
| pal (IBL16-03) * | ∆*pal*:: *cat* JM109(DE3) strain | In this study | | |
| lpp (IBL16-04) * | ∆*lpp*:: *kan* JM109(DE3) strain | In this study | | |
| lpp mrcB (IBL16-05) * | ∆*lpp*, ∆*mrcB*:: *cat* JM109(DE3) strain | In this study | | |
| mrcA lpp (IBL16-06) * | ∆*mrcA*, ∆*lpp*:: *kan* JM109(DE3) strain | In this study | | |
| lpp pal (IBL16-07) * | ∆*lpp*, ∆*pal*:: *kan* JM109(DE3) strain | In this study | | |
| mrcA pal (IBL16-08) * | ∆*mrcA*, ∆*pal*:: *cat* JM109(DE3) strain | In this study | | |
| mrcB pal (IBL16-09) * | ∆*mrcB*, ∆*pal*:: *cat* JM109(DE3) strain | In this study | | |
| mrcA N (IBL16-01N) * | ∆*mrcA* JM109(DE3) strain without *kan* | In this study | | |
| mrcB N (IBL16-02N) * | ∆*mrcB* JM109(DE3) strain without *kan* | In this study | | |
| lpp N (IBL16-04N) * | ∆*lpp* JM109(DE3) strain without *kan* | In this study | | |
| **Plasmid** | **Genotype** | **Source** | | |
| pKD3 | Cm^R^, Amp^R^, ori:R6K^γ^, source of cat cassette | | (Datsenko and Wanner, 2000) | |
| pKD4 | Kan^R^, Amp^R^, ori:R6K^γ^, source of *kan* cassette | | (Datsenko and Wanner, 2000) | |
| pKD 46 | Amp^R^, ParaB promoter, lambda-Red recombinase expression plasmid , ori:repA101ts | | (Datsenko and Wanner, 2000) | |
| pCP20 | Plasmid of expressing FLP recombinase  Cm^R^, Amp^R^, ori:repA101ts | | (Datsenko and Wanner, 2000) | |
| pET32a-*pth* | Amp^R^, T7 promoter, pBR322 origin, *pth* cloned into pET32a | | Our lab | |
| pET22b-*rpa* | Amp^R^, T7 promoter, pBR322 origin, *rpa* cloned into pET22b | | Our lab | |
| **Primers** | **Primer sequence (5´ to 3´)** | | | **Note** |
| mrcA-F | gcgtttgtttataaactgcccaaatgaaactaaatgggaaatttccagtggtgtaggctggagctgcttc | | | ** |
| mrcA-R | actgaaaaggcgccgaagcgcctttttaatcagaacaattcctgtgcctcatgggaattagccatggtcc | | | ** |
| mrcA-vF | ctgcccaaatgaaactaaatg | | | *** |
| mrcA-vR | ttgctggaagaccctgact | | | *** |
| mrcB-F | agaacagaaaatcgggcttttgcgcctgaatattgcggagaaaaagcatggtgtaggctggagctgcttc | | | ** |
| mrcB-R | ctcgccatccggtatttcacgcttagatgttaattactaccaaacatatcatgggaattagccatggtcc | | | ** |
| mrcB-vF | tcgagcacaaattttgagag | | | *** |
| mrcB-vR | caaccagatgaaaagaaagg | | | *** |
| lpp-F | acttgtaacgctacatggagattaactcaatctagagggtattaataatggtgtaggctggagctgcttc | | | ** |
| lpp-R | gtgcgccatttttcacttcacaggtactattacttgcggtatttagtagcatgggaattagccatggtcc | | | ** |
| lpp-vF | cagcgttcgatgcttcttt | | | *** |
| lpp-vR | gtttcggtgagcagtggag | | | *** |
| pal-F | gccgtatctgtgataataattaattgaatagtaaaggaatcattgaaatggtgtaggctggagctgcttc | | | ** |
| pal-R | tgtctgaagttactgctcatgcaattctcttagtaaaccagtaccgcacgatgggaattagccatggtcc | | | ** |
| pal-vF | aagtgctgaaagggctgat | | | *** |
| pal-vR | aagttgttgctggagttgg | | | *** |

* Strain serial number in our laboratory

** The underlined sequence represents homologous region flanked to the targeted gene

*** The primer couples mrcA-vF/mrcA-vR, mrcB-vF/mrcB-vR, lpp-vF/lpp-vR and pal-vF/pal-vR were used for verifying leaky strains with deleted genes *mrcB*, *mrcA*, *lpp* and *pal*, respectively.

Table S2. The sequences of TFs (targeting fragments)

| TFs | Sequences | Size |
| --- | --- | --- |
| mrcA -*kan* TF | gcgtttgtttataaactgcccaaatgaaactaaatgggaaatttccagtggtgtaggctggagctgcttcGAAGTTCCTATACTTTCTAGAGAATAGGAACTTCGGAATAGGAACTTCaagatcccctcacgctgccgcaagcactcagggcgcaagggctgctaaaggaagcggaacacgtagaaagccagtccgcagaaacggtgctgaccccggatgaatgtcagctactgggctatctggacaagggaaaacgcaagcgcaaagagaaagcaggtagcttgcagtgggcttacatggcgatagctagactgggcggttttatggacagcaagcgaaccggaattgccagctggggcgccctctggtaaggttgggaagccctgcaaagtaaactggatggctttcttgccgccaaggatctgatggcgcaggggatcaagatctgatcaagagacaggatgaggatcgtttcgcatgattgaacaagatggattgcacgcaggttctccggccgcttgggtggagaggctattcggctatgactgggcacaacagacaatcggctgctctgatgccgccgtgttccggctgtcagcgcaggggcgcccggttctttttgtcaagaccgacctgtccggtgccctgaatgaactgcaggacgaggcagcgcggctatcgtggctggccacgacgggcgttccttgcgcagctgtgctcgacgttgtcactgaagcgggaagggactggctgctattgggcgaagtgccggggcaggatctcctgtcatctcaccttgctcctgccgagaaagtatccatcatggctgatgcaatgcggcggctgcatacgcttgatccggctacctgcccattcgaccaccaagcgaaacatcgcatcgagcgagcacgtactcggatggaagccggtcttgtcgatcaggatgatctggacgaagagcatcaggggctcgcgccagccgaactgttcgccaggctcaaggcgcgcatgcccgacggcgaggatctcgtcgtgacccatggcgatgcctgcttgccgaatatcatggtggaaaatggccgcttttctggattcatcgactgtggccggctgggtgtggcggaccgctatcaggacatagcgttggctacccgtgatattgctgaagagcttggcggcgaatgggctgaccgcttcctcgtgctttacggtatcgccgctcccgattcgcagcgcatcgccttctatcgccttcttgacgagttcttctgagcgggactctggggttcgaaatgaccgaccaagcgacgcccaacctgccatcacgagatttcgattccaccgccgccttctatgaaaggttgggcttcggaatcgttttccgggacgccggctggatgatcctccagcgcggggatctcatgctggagttcttcgcccaccccagcttcaaaagcgctctGAAGTTCCTATACTTTCTAGAGAATAGGAACTTCGGAATAGGAACTaaggaggatattcatatggaccatggctaattcccatgaggcacaggaattgttctgattaaaaaggcgcttcggcgccttttcagt | 1596 |
| mrcB -*kan* TF | agaacagaaaatcgggcttttgcgcctgaatattgcggagaaaaagcatggtgtaggctggagctgcttcGAAGTTCCTATACTTTCTAGAGAATAGGAACTTCGGAATAGGAACTTCaagatcccctcacgctgccgcaagcactcagggcgcaagggctgctaaaggaagcggaacacgtagaaagccagtccgcagaaacggtgctgaccccggatgaatgtcagctactgggctatctggacaagggaaaacgcaagcgcaaagagaaagcaggtagcttgcagtgggcttacatggcgatagctagactgggcggttttatggacagcaagcgaaccggaattgccagctggggcgccctctggtaaggttgggaagccctgcaaagtaaactggatggctttcttgccgccaaggatctgatggcgcaggggatcaagatctgatcaagagacaggatgaggatcgtttcgcatgattgaacaagatggattgcacgcaggttctccggccgcttgggtggagaggctattcggctatgactgggcacaacagacaatcggctgctctgatgccgccgtgttccggctgtcagcgcaggggcgcccggttctttttgtcaagaccgacctgtccggtgccctgaatgaactgcaggacgaggcagcgcggctatcgtggctggccacgacgggcgttccttgcgcagctgtgctcgacgttgtcactgaagcgggaagggactggctgctattgggcgaagtgccggggcaggatctcctgtcatctcaccttgctcctgccgagaaagtatccatcatggctgatgcaatgcggcggctgcatacgcttgatccggctacctgcccattcgaccaccaagcgaaacatcgcatcgagcgagcacgtactcggatggaagccggtcttgtcgatcaggatgatctggacgaagagcatcaggggctcgcgccagccgaactgttcgccaggctcaaggcgcgcatgcccgacggcgaggatctcgtcgtgacccatggcgatgcctgcttgccgaatatcatggtggaaaatggccgcttttctggattcatcgactgtggccggctgggtgtggcggaccgctatcaggacatagcgttggctacccgtgatattgctgaagagcttggcggcgaatgggctgaccgcttcctcgtgctttacggtatcgccgctcccgattcgcagcgcatcgccttctatcgccttcttgacgagttcttctgagcgggactctggggttcgaaatgaccgaccaagcgacgcccaacctgccatcacgagatttcgattccaccgccgccttctatgaaaggttgggcttcggaatcgttttccgggacgccggctggatgatcctccagcgcggggatctcatgctggagttcttcgcccaccccagcttcaaaagcgctctGAAGTTCCTATACTTTCTAGAGAATAGGAACTTCGGAATAGGAACTaaggaggatattcatatggaccatggctaattcccatgatatgtttggtagtaattaacatctaagcgtgaaataccggatggcgag | 1596 |
| pal -*kan* TF | gccgtatctgtgataataattaattgaatagtaaaggaatcattgaaatggtgtaggctggagctgcttcGAAGTTCCTATACTTTCTAGAGAATAGGAACTTCGGAATAGGAACTTCaagatcccctcacgctgccgcaagcactcagggcgcaagggctgctaaaggaagcggaacacgtagaaagccagtccgcagaaacggtgctgaccccggatgaatgtcagctactgggctatctggacaagggaaaacgcaagcgcaaagagaaagcaggtagcttgcagtgggcttacatggcgatagctagactgggcggttttatggacagcaagcgaaccggaattgccagctggggcgccctctggtaaggttgggaagccctgcaaagtaaactggatggctttcttgccgccaaggatctgatggcgcaggggatcaagatctgatcaagagacaggatgaggatcgtttcgcatgattgaacaagatggattgcacgcaggttctccggccgcttgggtggagaggctattcggctatgactgggcacaacagacaatcggctgctctgatgccgccgtgttccggctgtcagcgcaggggcgcccggttctttttgtcaagaccgacctgtccggtgccctgaatgaactgcaggacgaggcagcgcggctatcgtggctggccacgacgggcgttccttgcgcagctgtgctcgacgttgtcactgaagcgggaagggactggctgctattgggcgaagtgccggggcaggatctcctgtcatctcaccttgctcctgccgagaaagtatccatcatggctgatgcaatgcggcggctgcatacgcttgatccggctacctgcccattcgaccaccaagcgaaacatcgcatcgagcgagcacgtactcggatggaagccggtcttgtcgatcaggatgatctggacgaagagcatcaggggctcgcgccagccgaactgttcgccaggctcaaggcgcgcatgcccgacggcgaggatctcgtcgtgacccatggcgatgcctgcttgccgaatatcatggtggaaaatggccgcttttctggattcatcgactgtggccggctgggtgtggcggaccgctatcaggacatagcgttggctacccgtgatattgctgaagagcttggcggcgaatgggctgaccgcttcctcgtgctttacggtatcgccgctcccgattcgcagcgcatcgccttctatcgccttcttgacgagttcttctgagcgggactctggggttcgaaatgaccgaccaagcgacgcccaacctgccatcacgagatttcgattccaccgccgccttctatgaaaggttgggcttcggaatcgttttccgggacgccggctggatgatcctccagcgcggggatctcatgctggagttcttcgcccaccccagcttcaaaagcgctctGAAGTTCCTATACTTTCTAGAGAATAGGAACTTCGGAATAGGAACTaaggaggatattcatatggaccatggctaattcccatcgtgcggtactggtttactaagagaattgcatgagcagtaacttcagaca | 1596 |
| lpp -*kan* TF | acttgtaacgctacatggagattaactcaatctagagggtattaataatggtgtaggctggagctgcttcGAAGTTCCTATACTTTCTAGAGAATAGGAACTTCGGAATAGGAACTTCaagatcccctcacgctgccgcaagcactcagggcgcaagggctgctaaaggaagcggaacacgtagaaagccagtccgcagaaacggtgctgaccccggatgaatgtcagctactgggctatctggacaagggaaaacgcaagcgcaaagagaaagcaggtagcttgcagtgggcttacatggcgatagctagactgggcggttttatggacagcaagcgaaccggaattgccagctggggcgccctctggtaaggttgggaagccctgcaaagtaaactggatggctttcttgccgccaaggatctgatggcgcaggggatcaagatctgatcaagagacaggatgaggatcgtttcgcatgattgaacaagatggattgcacgcaggttctccggccgcttgggtggagaggctattcggctatgactgggcacaacagacaatcggctgctctgatgccgccgtgttccggctgtcagcgcaggggcgcccggttctttttgtcaagaccgacctgtccggtgccctgaatgaactgcaggacgaggcagcgcggctatcgtggctggccacgacgggcgttccttgcgcagctgtgctcgacgttgtcactgaagcgggaagggactggctgctattgggcgaagtgccggggcaggatctcctgtcatctcaccttgctcctgccgagaaagtatccatcatggctgatgcaatgcggcggctgcatacgcttgatccggctacctgcccattcgaccaccaagcgaaacatcgcatcgagcgagcacgtactcggatggaagccggtcttgtcgatcaggatgatctggacgaagagcatcaggggctcgcgccagccgaactgttcgccaggctcaaggcgcgcatgcccgacggcgaggatctcgtcgtgacccatggcgatgcctgcttgccgaatatcatggtggaaaatggccgcttttctggattcatcgactgtggccggctgggtgtggcggaccgctatcaggacatagcgttggctacccgtgatattgctgaagagcttggcggcgaatgggctgaccgcttcctcgtgctttacggtatcgccgctcccgattcgcagcgcatcgccttctatcgccttcttgacgagttcttctgagcgggactctggggttcgaaatgaccgaccaagcgacgcccaacctgccatcacgagatttcgattccaccgccgccttctatgaaaggttgggcttcggaatcgttttccgggacgccggctggatgatcctccagcgcggggatctcatgctggagttcttcgcccaccccagcttcaaaagcgctctGAAGTTCCTATACTTTCTAGAGAATAGGAACTTCGGAATAGGAACTaaggaggatattcatatggaccatggctaattcccatgctactaaataccgcaagtaatagtacctgtgaagtgaaaaatggcgcac | 1596 |
| mrcA -*cat* TF | gcgtttgtttataaactgcccaaatgaaactaaatgggaaatttccagtggtgtaggctggagctgcttcGAAGTTCCTATACTTTCTAGAGAATAGGAACTTCGGAATAGGAACTTCatttaaatggcgcgccttacgccccgccctgccactcatcgcagtactgttgtattcattaagcatctgccgacatggaagccatcacaaacggcatgatgaacctgaatcgccagcggcatcagcaccttgtcgccttgcgtataatatttgcccatggtgaaaacgggggcgaagaagttgtccatattggccacgtttaaatcaaaactggtgaaactcacccagggattggctgagacgaaaaacatattctcaataaaccctttagggaaataggccaggttttcaccgtaacacgccacatcttgcgaatatatgtgtagaaactgccggaaatcgtcgtggtattcactccagagcgatgaaaacgtttcagtttgctcatggaaaacggtgtaacaagggtgaacactatcccatatcaccagctcaccgtctttcattgccatacgtaattccggatgagcattcatcaggcgggcaagaatgtgaataaaggccggataaaacttgtgcttatttttctttacggtctttaaaaaggccgtaatatccagctgaacggtctggttataggtacattgagcaactgactgaaatgcctcaaaatgttctttacgatgccattgggatatatcaacggtggtatatccagtgatttttttctccattttagcttccttagctcctgaaaatctcgacaactcaaaaaatacgcccggtagtgatcttatttcattatggtgaaagttggaacctcttacgtgccgatcaacgtctcattttcgccaaaagttggcccagggcttcccggtatcaacagggacaccaggatttatttattctgcgaagtgatcttccgtcacaggtaggcgcgccGAAGTTCCTATACTTTCTAGAGAATAGGAACTTCGGAATAGGAACTaaggaggatattcatatggaccatggctaattcccatgaggcacaggaattgttctgattaaaaaggcgcttcggcgccttttcagt | 1133 |
| mrcB -*cat* TF | agaacagaaaatcgggcttttgcgcctgaatattgcggagaaaaagcatggtgtaggctggagctgcttcGAAGTTCCTATACTTTCTAGAGAATAGGAACTTCGGAATAGGAACTTCatttaaatggcgcgccttacgccccgccctgccactcatcgcagtactgttgtattcattaagcatctgccgacatggaagccatcacaaacggcatgatgaacctgaatcgccagcggcatcagcaccttgtcgccttgcgtataatatttgcccatggtgaaaacgggggcgaagaagttgtccatattggccacgtttaaatcaaaactggtgaaactcacccagggattggctgagacgaaaaacatattctcaataaaccctttagggaaataggccaggttttcaccgtaacacgccacatcttgcgaatatatgtgtagaaactgccggaaatcgtcgtggtattcactccagagcgatgaaaacgtttcagtttgctcatggaaaacggtgtaacaagggtgaacactatcccatatcaccagctcaccgtctttcattgccatacgtaattccggatgagcattcatcaggcgggcaagaatgtgaataaaggccggataaaacttgtgcttatttttctttacggtctttaaaaaggccgtaatatccagctgaacggtctggttataggtacattgagcaactgactgaaatgcctcaaaatgttctttacgatgccattgggatatatcaacggtggtatatccagtgatttttttctccattttagcttccttagctcctgaaaatctcgacaactcaaaaaatacgcccggtagtgatcttatttcattatggtgaaagttggaacctcttacgtgccgatcaacgtctcattttcgccaaaagttggcccagggcttcccggtatcaacagggacaccaggatttatttattctgcgaagtgatcttccgtcacaggtaggcgcgccGAAGTTCCTATACTTTCTAGAGAATAGGAACTTCGGAATAGGAACTaaggaggatattcatatggaccatggctaattcccatgatatgtttggtagtaattaacatctaagcgtgaaataccggatggcgag | 1133 |
| pal -*cat* TF | gccgtatctgtgataataattaattgaatagtaaaggaatcattgaaatggtgtaggctggagctgcttcGAAGTTCCTATACTTTCTAGAGAATAGGAACTTCGGAATAGGAACTTCatttaaatggcgcgccttacgccccgccctgccactcatcgcagtactgttgtattcattaagcatctgccgacatggaagccatcacaaacggcatgatgaacctgaatcgccagcggcatcagcaccttgtcgccttgcgtataatatttgcccatggtgaaaacgggggcgaagaagttgtccatattggccacgtttaaatcaaaactggtgaaactcacccagggattggctgagacgaaaaacatattctcaataaaccctttagggaaataggccaggttttcaccgtaacacgccacatcttgcgaatatatgtgtagaaactgccggaaatcgtcgtggtattcactccagagcgatgaaaacgtttcagtttgctcatggaaaacggtgtaacaagggtgaacactatcccatatcaccagctcaccgtctttcattgccatacgtaattccggatgagcattcatcaggcgggcaagaatgtgaataaaggccggataaaacttgtgcttatttttctttacggtctttaaaaaggccgtaatatccagctgaacggtctggttataggtacattgagcaactgactgaaatgcctcaaaatgttctttacgatgccattgggatatatcaacggtggtatatccagtgatttttttctccattttagcttccttagctcctgaaaatctcgacaactcaaaaaatacgcccggtagtgatcttatttcattatggtgaaagttggaacctcttacgtgccgatcaacgtctcattttcgccaaaagttggcccagggcttcccggtatcaacagggacaccaggatttatttattctgcgaagtgatcttccgtcacaggtaggcgcgccGAAGTTCCTATACTTTCTAGAGAATAGGAACTTCGGAATAGGAACTaaggaggatattcatatggaccatggctaattcccatcgtgcggtactggtttactaagagaattgcatgagcagtaacttcagaca | 1133 |
| lpp -*cat* TF | acttgtaacgctacatggagattaactcaatctagagggtattaataatggtgtaggctggagctgcttcGAAGTTCCTATACTTTCTAGAGAATAGGAACTTCGGAATAGGAACTTCatttaaatggcgcgccttacgccccgccctgccactcatcgcagtactgttgtattcattaagcatctgccgacatggaagccatcacaaacggcatgatgaacctgaatcgccagcggcatcagcaccttgtcgccttgcgtataatatttgcccatggtgaaaacgggggcgaagaagttgtccatattggccacgtttaaatcaaaactggtgaaactcacccagggattggctgagacgaaaaacatattctcaataaaccctttagggaaataggccaggttttcaccgtaacacgccacatcttgcgaatatatgtgtagaaactgccggaaatcgtcgtggtattcactccagagcgatgaaaacgtttcagtttgctcatggaaaacggtgtaacaagggtgaacactatcccatatcaccagctcaccgtctttcattgccatacgtaattccggatgagcattcatcaggcgggcaagaatgtgaataaaggccggataaaacttgtgcttatttttctttacggtctttaaaaaggccgtaatatccagctgaacggtctggttataggtacattgagcaactgactgaaatgcctcaaaatgttctttacgatgccattgggatatatcaacggtggtatatccagtgatttttttctccattttagcttccttagctcctgaaaatctcgacaactcaaaaaatacgcccggtagtgatcttatttcattatggtgaaagttggaacctcttacgtgccgatcaacgtctcattttcgccaaaagttggcccagggcttcccggtatcaacagggacaccaggatttatttattctgcgaagtgatcttccgtcacaggtaggcgcgccGAAGTTCCTATACTTTCTAGAGAATAGGAACTTCGGAATAGGAACTaaggaggatattcatatggaccatggctaattcccatgctactaaataccgcaagtaatagtacctgtgaagtgaaaaatggcgcac | 1133 |

The capitalized sequence is FRT site

The underlined sequence represents homologous region flanked to the targeted gene

Table S3. The plasmids, primers, resistance markers, targeting fragments and hosts used for mutant construct

| Mutants | Plasmids | Primers | Genetic markers | Targeting fragments | Hosts |
| --- | --- | --- | --- | --- | --- |
| mrcA | pKD4 | mrcA-F, mrcA-R  mrcA-vF, mrcA-vR | *kan* | mrcA-*kan* TF | JM109 (DE3) -pKD46 |
| mrcB | pKD46  pKD4 | mrcB-F, mrcB-R  mrcB-vF, mrcB-vR | *kan* | mrcB-*kan* TF | JM109 (DE3) -pKD46 |
| pal | pKD46  pKD3 | pal-F, pal-R  pal-vF, pal-vR | *cat* | pal- *cat* TF | JM109 (DE3) -pKD46 |
| lpp | pKD46  pKD4 | lpp-F, lpp -R  lpp -vF, lpp -vR | *kan* | lpp- *kan* TF | JM109 (DE3) -pKD46 |
| mrcA N | pCP20 |  | - |  | mrcA |
| mrcB N | pCP20 |  | - |  | mrcB |
| lpp N | pCP20 |  | - |  | lpp |
| lpp mrcB | pKD46, pKD3 | mrcB-F, mrcB-R  mrcB-vF, mrcB-vR | *cat* | mrcB-*cat* TF | lpp–pKD46 |
| mrcA lpp | pKD46  pKD4 | mrcA-F, mrcA-R  mrcA-vF, mrcA-vR | *kan* | mrcA-*kan* TF | mrcA -pKD46 |
| lpp pal | pKD46  pKD4 | pal-F, pal-R  pal-vF, pal-vR | *kan* | pal- *kan* TF | lpp-pKD46 |
| mrcA pal | pKD46  pKD3 | pal-F, pal-R  pal-vF, pal-vR | *cat* | pal- *cat* TF | mrcA -pKD46 |
| mrcB pal | pKD46  pKD3 | pal-F, pal-R  pal-vF, pal-vR | *cat* | pal- *cat* TF | mrcB -pKD46 |
| JM109(DE3)-*pth* (IBL16-*pth*)* | pET32a-*pth* |  | *amp* |  | JM109 (DE3) |
| mrcA-*pth* (IBL16-01-*pth*)* | pET32a-*pth* |  | *kan*, *bla*(Amp^R^) |  | mrcA |
| mrcB-*pth* (IBL16-02-*pth*)* | pET32a-*pth* |  | *kan*, *bla* |  | mrcB |
| pal-*pth* (IBL16-03-*pth*)* | pET32a-*pth* |  | *cat*, *bla* |  | pal |
| lpp-*pth* (IBL16-04-*pth*)* | pET32a-*pth* |  | *kan*, *bla* |  | lpp |
| lpp mrcB-*pth* (IBL16-05-*pth*)* | pET32a-*pth* |  | *cat*, *bla* |  | lpp mrcB |
| mrcA lpp-*pth* (IBL16-06-*pth*)* | pET32a-*pth* |  | *kan*, *bla* |  | mrcA lpp |
| lpp pal-*pth* (IBL16-07-*pth*)* | pET32a-*pth* |  | *kan*, *bla* |  | lpp pal |
| mrcA pal-*pth* (IBL16-08-*pth*)* | pET32a-*pth* |  | *cat*, *bla* |  | mrcA pal |
| mrcB pal-*pth* (IBL16-09-*pth*)* | pET32a-*pth* |  | *cat*, *bla* |  | mrcB pal |
| JM109 (DE3) – *rpa* (IBL16-*rpa*)* | pET22b-*rpa* |  | *bla* |  | JM109 (DE3) |
| lpp mrcB-*rpa* (IBL16-05-*rpa*)* | pET22b-*rpa* |  | *cat*, *bla* |  | lpp mrcB |

* Mutant serial number in our laboratory

Table S4. Sequences of the PCR products of the mutants of mrcAN, mrcBN and lppN.

| strains | Sequence |
| --- | --- |
| mrcA N | tctgcccaaatgaaactaaatgggaaatttccaGTGgtgtaggctggagctgcttcgaagttcctatactttctagagaataggaacttcggaataggaactaaggaggatattcatatggaccatggctaattcccatgaggcacaggaattgttcTGAttaaaaaggcgcttcggcgccttttcagtttgctgacaaagtgcacttgtttatgccggatacggcgtgaacgcgttatccggctaacaaaatcgtgaaaactcaataaattgcagaaaccccataagcctgataaacattgtgcatcaggcaaacttcacgcatttacactcgcccctgcccttcaaccattcgcgcacgagaacagcgcactgacaatgccgtt |
| mrcB N | atagactgtaagtagcacagatatcgggcttttgcgcctgaatattgcggagaaaagcATGgtgtaggctggagctgcttcgaagttcctatactttctagagaataggaacttcggaataggaactaaggaggatattcatatggaccatggctaattcccatgatatgtttggtagtaatTAAcatctaagcgtgaaataccggatggcgagttgccatccggtaaaataacatcccatctaagatattaaccctttcttttcaatctggttga |
| lpp N | tcagcgttcgatgcttctttgagcgaacgatcaaaaataagtgccttcccatcaaaaaaatattctcaacataaaaaactttgtgtaatacttgtaacgctacatggagattaactcaatctagagggtattaataATGgtgtaggctggagctgcttcgaagttcctatactttctagagaataggaacttcggaataggaactaaggaggatattcatatggaccatggctaattcccatgctactaaataccgcaagTAAttgtacctgtgaagtgaaaaatggcgcacattgtgcgccattttttttgtctgccgtttaccgctactgcgtcacgcgtaacatattcccttgctctggttcaccattctgcgctgactctacgaagacgcattgcggtttcggg |

The capitalized sequence is initiation codon or stop codon of targeted genes.

Table S5. Growth analysis and protein detection from the recombinant leaky strains with single gene deletion in TB medium.

| Strains | Final OD_600_ | Trx-hPTH | | | Total proteins | |
| --- | --- | --- | --- | --- | --- | --- |
|  |  | EPS (mg/l) | IPS (mg/l) | (mg/l) | | |
| mrcA-*pth* | 12.80 | -^*^ | 610 ± 10 | 2560 ± 60 | | |
| mrcB-*pth* | 12.50 | - | 500 ± 10 | 2310 ± 60 | | |
| pal-*pth* | 8.70 | 250 ± 170 | 320 ± 2 | 2230 ± 410 | | |
| lpp-*pth* | 9.98 | 270 ± 100 | 430 ± 10 | 2380 ± 210 | | |
| JM109 (DE3)- *pth* | 14.90 | - | 530 ± 20 | | | 5070 ± 300 |
| JM109 (DE3) | 13.38 | - | - | 2910 ± 120 | | |

^*^. Not detected**.**

Protein concentrations are expressed as mean ± SEM of n=2.

Table S6. Growth analysis and protein detection from the recombinant leaky strains with double deletion in TB medium.

| Strains | Final OD_600_ | Trx-hPTH | | Total proteins | | |
| --- | --- | --- | --- | --- | --- | --- |
|  |  | EPS (mg/l) | IPS (mg/l) | | (mg/l) | |
| lpp mrcB-*pth* | 5.52 | 510 ± 140 | 220 ± 1 | | 344 ± 630 | |
| mrcA lpp-*pth* | 10.82 | 540 ± 4 | 350 ± 20 | | 4040 ± 80 | |
| lpp pal-*pth* | 9.38 | 420 ± 150 | 160 ± 1 | | 3360 ± 700 | |
| mrcA pal-*pth* | 6.26 | 330 ± 180 | 190 ± 10 | | 2250 ± 640 | |
| mrcB pal-*pth* | 8.74 | 330 ± 140 | 280 ± 10 | | 2430 ± 470 | |
| JM109 (DE3)- *pth* | 13.04 | -^*^ | 500 ± 10 | | | 4460 ± 500 |
| JM109 (DE3) | 12.46 | - | - | | 4260 ± 200 | |

^*.^Not detected.

The protein concentrations are expressed as Mean ± SEM of n=2.

Fig. S1. Verification of the mutants by colony PCR (Lane M: representing standard marker). (A) Single knockout of gene *mrcA* (*kan*/1700 bp): Lane 1, JM109 (DE3) as a template; Lane 2, 3 and 4, mrcA as a template; (B) Single knockout of gene *mrcB* (*kan*/1700 bp): Lane 1 and 2, JM109 (DE3) as a template; Lane 3 and 4, mrcB as a template; (C) Single knockout of gene *pal* (*cat*/1600 bp): Lane 1 and 2, JM109 (DE3) as a template; Lane 3, 4 and 5, pal as a template; (D) Single knockout of gene *lpp* (*kan*/1800 bp): Lane 1, 2, 3 and 4, lpp N as a template; Lane5 JM109 (DE3) as a template; Lane 6, 7 and 8, lpp as a template; (E) Double knockout of genes *lpp* and *mrcB* (*cat*/1200 bp) as well as *mrcA* and *lpp* (*kan*/1800 bp); Lane1 and 6, others, Lane 3, JM109 (DE3) as a template; Lane 4, lpp mrcB (Cm^R^) as a template (mrcB-vF and mrcB-vR as primers); Lane 5, lpp mrcB (Kan^R^) as a template (mrcB-vF and mrcB-vR as primers); Lane 6, lpp N as a template (lpp-vF and lpp-vR as primers); Lane 7, mrcA lpp (Kan^R^) as a template (lpp-vF and lpp-vR as primers); (F) Double knockout of genes *lpp* and *pal* (*kan /*2100 bp); Lane 1 and 2, lpp pal (Kan^R^) as a template (pal-vF and pal-vR as primers); Lane 3and 4, (Cm^R^) as a template (pal-vF and pal-vR as primers); Lane 5, nothing; lane 6, JM109 (DE3) as a template; (G) Double knockout of genes *pal* and *mrcB* (*kan* /1700 bp) as well as *pal* and *mrcA* (*kan*/1700 bp); Lane 1, 3 and 5, mrcB pal as a template (pal-vF and pal-vR as primers); Lane 2 and 4, false positive colonies as a template (pal-vF and pal-vR as primers); Lane 6, 7, 8 and 9, JM109 (DE3) as a template; Lane 10, mrcA pal as a template (pal-vF and pal-vR as primers).

Fig. S2. SDS PAGE analysis of protein samples in TB medium. Lane 1 and 2, the extracellular proteins of the recombinant leaky strains lpp mrcB with pET32a-*pth*; Lane 3, 4, 5 and 6, the extracellular proteins of the recombinant leaky strains mrcA lpp, lpp, mrcB and mrcA with pET32a-*pth*; Lane 7, the extracellular proteins of the strains JM109 (DE3) harboring pET32a-*pth*; M, protein ladder; Lane 8, 9, 10, 11, 12, 13 and 14, intracellular soluble proteins corresponding to lane 1, 2, 3, 4, 5 , 6 and 7.

Fig. S3. SDS-PAGE analysis of protein samples of lpp mrcB with pET22b-*rpa* in TB medium (M, protein ladder). Lane 1, 2, 3, and 4, the extracellular proteins of the recombinant strains lpp mrcB, mrcB, lpp and JM109 (DE3) harboring pET22b-*rpa*; Lane 5, 6, 7 and 8, intracellular soluble proteins corresponding to lane 1, 2, 3 and 4; Lane 9, 10, 11 and 12, intracellular insoluble proteins corresponding to lane 1, 2, 3 and 4.

(A)

(B)

Fig. S4. Growth curve of the strains in flasks in LB medium.(A)■, strain lpp mrcB with pET32a-*pth*; ●, Strain lpp mrcB harboring pET32a-*pth* with IPTG induction after four hour cultivation ; ▲, Strain mrcA lpp with pET32a-*pth*. (B) ■, strain mrcB; ●, strain JM109(DE3).
